# The Relationship Between Shoulder Intra-Articular Joint Pressure Change, Operative Variables, Early Postoperative Pain and Opioid Consumption: A Pilot Study

**DOI:** 10.7759/cureus.104313

**Published:** 2026-02-26

**Authors:** Nathan Li, Drew R Dalby, Kai Zhu, Johnny Kasto, Christopher Nikolopoulos, Jared M Mahylis, Stephanie Muh

**Affiliations:** 1 Department of Orthopaedic Surgery, Henry Ford Health System, Detroit, USA

**Keywords:** arthroscopy, compartment pressure, joint pressure, opioid consumption, postoperative pain, shoulder

## Abstract

Introduction: This pilot study evaluated the relationship between intra-articular joint pressure changes and fluid volume during shoulder arthroscopy, relating these to postoperative pain and opioid consumption.

Methods: Fifty-two patients who underwent shoulder arthroscopic procedures were included. Measurements of joint pressure, fluid volume utilized, and postoperative opioid intake were recorded. Pain levels were assessed using the visual analog scale preoperatively and one week postoperatively. Pressure change was measured as postoperative minus preoperative intra-articular pressure.

Results: Initial mean joint pressure was 4.63 mm Hg, increasing to a mean of 33.9 mm Hg postoperatively. A negative correlation was observed between increased joint pressure change and visual analog scale delta (r = -0.23; *P* = 0.047), along with a positive correlation between increased joint pressure change and morphine milligram equivalents of opioids (r = 0.30; *P* = 0.02).

Conclusion: This exploratory study demonstrates that smaller changes in joint pressure correlate with a decrease in postoperative pain. Conversely, an increase in joint pressure was associated with heightened opioid consumption, demonstrating that maintaining low pressure can provide possible benefits with a decrease in postoperative pain and opioid consumption.

## Introduction

Since its inception, arthroscopic rotator cuff repair has become the gold standard in rotator cuff repair surgeries [1,2]. Its introduction decreased the rate of many complications associated with open rotator cuff repair, such as increased pain, infection risk, and deltoid avulsion [3-5]. During shoulder arthroscopic surgery, increasing the arthroscopic infusion pressure allows for greater joint distension and thus, greater visualization [6]. However, surgeons must also be cognizant of avoiding excessive shoulder pressure that can lead to life-threatening fluid extravasation in rare situations [7], which occurs when the fluid leaks into the surrounding tissue and can lead to respiratory distress and neurologic complications [8,9].

Controlling postoperative pain following arthroscopic surgery continues to be an essential part of patient care. Postoperative pain is multifactorial, including patient-specific factors that can be difficult to predict. Unpredicted pain can lead to a negative impact on patient experience and quality of life in the immediate postoperative period [10]. Factors such as sensitivity to pain [11] and patient perception of shoulder function [12] have been shown to affect postoperative pain. Current literature has explored many preoperative variables involved in postoperative pain, including high initial visual analog scale (VAS) scores, acute onset of pain, and psychosocial characteristics [13-16]. To our knowledge, there has yet to be any previous literature on the association of intra-articular shoulder pressure with postoperative pain perception.

The aim of this pilot study was to evaluate the correlation between changes in intra-articular shoulder joint pressure and volume of fluid used during shoulder arthroscopic surgery with early postoperative pain and postoperative opioid consumption. We hypothesized that a larger change in intra-articular joint pressure would be associated with worse postoperative pain and increased opioid consumption.

## Materials and methods

Following institutional review board approval, prospective enrollment of 52 patients undergoing shoulder arthroscopic surgery from July 2021 to November 2023 at a single institution was performed. Informed consent for study participation was obtained pre-operatively during office visits. All surgeries were performed by a single fellowship-trained orthopedic surgeon. Inclusion criteria included patients at least 18 years of age who had an arthroscopic intervention for the treatment of rotator cuff tear, bicipital tenosynovitis, adhesive capsulitis, impingement syndrome, and shoulder instability. Patients were excluded if they had previous ipsilateral shoulder surgery, revision surgery, infections, were younger than 18 years old, were chronic opioid users [17], were currently taking blood thinner medications, or underwent surgery other than shoulder arthroscopy.

Peri-procedural intra-articular pressure was assessed directly using the STIC Intra-Compartmental Pressure Monitor System produced by C2Dx (Stryker Surgical, Kalamazoo, MI) [18]. Prior to the procedure, the device was primed and calibrated to the manufacturer's specifications. The surgeon inserted the intra-compartmental pressure monitoring needle into the patient’s glenohumeral joint, where three consecutive pressure measurements were obtained prior to the introduction of arthroscopic equipment. After the surgery was completed, the intra-articular pressure was measured three times again. The measurements were averaged into mean preoperative and postoperative shoulder pressure measurements. Subsequently, the mean change in shoulder pressure was calculated between the two datasets.

Preoperative pain was measured at the last clinic visit before surgery, and early postoperative pain consisted of a single VAS taken at the one-week follow-up appointment. Morphine milligram equivalents (MME) were determined by the number of opioids prescribed postoperatively with an indication of “shoulder pain” within a 90-day period after the date of surgery. It was calculated using conversion factors provided in the Utah Department of Health and Human Services opioid morphine milligram equivalent conversion factors [19]. The sample size for this study was determined to be approximately 50-60 patients. This decision was made based on a literature review, which revealed only one arguably similar study [20]. Due to the limited availability of relevant literature and the exploratory nature of this research, a formal power analysis was not conducted as this was a pilot study. Consequently, this study was intended to be descriptive in nature. Patient demographics, including age, race, sex, body mass index, and laterality, were also recorded. Race and sex were extracted from what patients reported within the electronic medical record. Race was categorized as either White, Black, or other. “Other” was defined as either Asian, Hispanic or Other. The term sex was classified into male or female based on biological distinction [21].

A Pearson correlation coefficient was performed to identify the strength and correlation between the change in intra-articular joint pressure and volume of fluid used with postoperative mean VAS, VAS delta, and MME. Linear regression analysis was used to explore the relationship between these intraoperative variables and postoperative pain scores and MME consumption. Multivariate analysis was performed to adjust for any potential confounders. Statistical significance was set at P < 0.05. All analyses were performed using IBM SPSS Statistics (Version 29.0, IBM Corp, Armonk, NY).

## Results

The cohort comprised 26 male patients and 26 female patients, with a mean age of 57.8 ± 9.0 years, a mean body mass index of 30.7 ± 6.1 kg per meter squared and a racial distribution of 48% White (n=28), 35% Black (n=18), and 11% Other (n=6). The most common preoperative diagnosis and procedure performed was rotator cuff tear (88%) n=46 and rotator cuff repair (88%) n=46. The mean preoperative pain score on the VAS was 6.2± 2.3, and the postoperative mean VAS was 3.62 ± 2.51, with a mean delta between preoperative and postoperative scores of -2.56 ± 2.53. The mean pre-surgical intra-articular pressure was 4.63 ± 5.87 mm Hg, and the mean postoperative pressure was 33.87 ± 15.56 mm Hg for a mean pressure change of 29.26 ± 16.71 mm Hg (Table 1).

**Table 1:** Descriptive statistics for demographics, intraoperative variables, and postoperative outcomes MME, morphine milligram equivalents; VAS, visual analog scale. Values are represented as mean ± standard deviation (range).

Patient Characteristics	All Patients (N=52)
Age (years), mean ± SD	57.8 ± 9.0
Body mass index (kg/m^2^), mean ± SD	30.7 ± 6.1
Sex, n (%)	
Female	26 (50%)
Male	26 (50%)
Race, n (%)	
White	28 (48%)
Black	18 (35%)
Other	6 (11%)
Laterality, n (%)	
Right	33 (64%)
Left	19 (36%)
Preoperative diagnosis, n (%)	
Rotator cuff tear	46 (88%)
Bicipital tenosynovitis	3 (6%)
Adhesive capsulitis	1 (2%)
Impingement syndrome	1 (2%)
Shoulder instability	1 (2%)
Arthroscopic procedure, n (%)	
Rotator cuff repair	46 (88%)
Arthroscopic debridement	4 (8%)
Bankart repair	1 (2%)
Arthroscopic capsular release	1 (2%)
Intraoperative variable and outcomes	
Preoperative VAS, mean ± SD (range)	6.17 ± 2.26 (1-10)
Postoperative VAS, mean ± SD (range)	3.62 ± 2.51 (0-10)
VAS delta, mean ± SD (range)	-2.56 ± 2.53 (-2 to 8)
MME, mean ± SD (range)	330.29 ± 232.98 (0 to 1530)
Preoperative pressure (mm Hg), mean ± SD (range)	4.63 ± 5.87 (-5.3 to 22.7)
Postoperative pressure (mm Hg), mean ± SD (range)	33.87 ± 15.56 (4.3-78)
Change in pressure (mm Hg), mean ± SD (range)	29.26 ± 16.71 (-3.3 to 75)

There was a weak but statistically significant negative correlation between increased intra-articular shoulder pressure and the delta between preoperative and postoperative VAS, or VAS delta (r = -0.23; P = 0.047). Patients who had a larger change in pressure experienced a smaller decrease in their post-op VAS compared to their pre-operative VAS score. This VAS delta will also be referred to as VAS-D. There was a significant positive correlation between the change in shoulder pressure and the dose of opioids (MME) postoperatively (r = 0.30; P = 0.02) (Table 2).

**Table 2:** Correlation analysis between intraoperative variables and postoperative pain and MME MME, morphine milligram equivalents; VAS, visual analog scale. *Indicates P-value < 0.05

Intraoperative Variables	Postoperative VAS		VAS Delta		MME	
	r	P-value	r	P-value	r	P-value
Change in pressure	0.10	0.24	-0.23	0.047^*^	0.30	0.02^*^
Amount of fluid	-0.12	0.20	-0.05	0.37	0.09	0.26

There was a significant linear relationship between intraoperative change in shoulder pressure and postoperative MME (P = 0.03). Specifically, for every one-unit increase in change in pressure, MME increased by 4.16 units (Table 3).

**Table 3:** Univariate linear regression analysis of intraoperative variables MME, morphine milligram equivalents; VAS, visual analog scale. *Indicates P-value < 0.05.

Intraoperative Variables	Postoperative VAS		VAS Delta		MME	
	Coefficient	P-value	Coefficient	P-value	Coefficient	P-value
Change in pressure	0.02	0.48	-0.04	0.1	4.16	0.03^*^
Amount of fluid	-0.06	0.40	-0.02	0.74	4.2	0.51

There was no significant multivariate linear relationship between intraoperative changes in shoulder pressure, postoperative MME, VAS, or VAS delta (Table 4).

**Table 4:** Multivariate linear regression analysis of intraoperative variables MME, morphine milligram equivalents; VAS, visual analog scale. *Indicates P-value < 0.05.

Intraoperative Variables	Postoperative VAS		VAS Delta		MME	
	Coefficient	P-value	Coefficient	P-value	Coefficient	P-value
Change in pressure	0.02	0.48	-0.03	0.17	3.00	0.14
Amount of fluid	-0.14	0.13	0.05	0.58	-10.30	0.19

## Discussion

Our results suggest that shoulder pressure during shoulder arthroscopic surgery is correlated with a patient's postoperative pain. Specifically, a higher change in joint pressure correlates with a smaller VAS delta and less improvement of their pain after surgery. Patients who experienced increased intra-articular joint pressure experienced significantly decreased relief of their preoperative pain compared to patients who did not experience increased shoulder pressure. One mechanism we suspect may have caused this increase in postoperative pain and narcotic use is the volume of fluid infusion and, subsequently, the volume of extravasated fluid. Stafford et al. found a significant increase in the volume of extravasated fluid and volume of fluid infused [22]. This coincides with our results, which showed that the volume of infusion is positively correlated with increased intra-articular pressure. Furthermore, Zhuang et al. found a negative correlation between volume of infusion and extravasation and decreased American Shoulder and Elbow Surgeons scores at three months postoperatively [23]. The American Shoulder and Elbow Surgeons score is used as a patient-reported metric with both pain and functional subscales [24], indicating a potential link. Interestingly, no difference was found between retention volume and VAS score 24 hours after the operation. However, the study did not measure the difference between preoperative and postoperative VAS. We suspect a similar mechanism of the escalation of postoperative pain and narcotics use, as shown by Tan et al. [20], who concluded that a higher arthroscopic infusion pressure of 80 mm Hg, rather than infusion volume, was a significant factor associated with greater postoperative pain after hip arthroscopy. There have been studies that have shown that intramuscular and intercompartmental pressures in the shoulder increase after arthroscopic surgery [9,25,26]. One study by Lee et al. demonstrated a deltoid intramuscular pressure of 48 mm Hg during arthroscopic subacromial decompression [27]. Subclinical tissue edema and the potential concern for compartment syndrome can arise when the intercompartmental pressure reaches a critical level, leading to poor perfusion and relative ischemia of the tissue. These factors could be considered contributing factors to pain [28]. When considering the linear relationship between changes in postoperative pain and MME usage, the association loses statistical significance in the multivariate logistical regression (P = 0.14), suggesting that the statistical significance observed in the linear regression (P = 0.03) may be due to confounding variables.

With these findings, our study adds valuable insights to the limited body of literature regarding the impact of intra-articular pressure during shoulder arthroscopy on postoperative pain and opioid consumption. Our results suggest the importance of surgeons recognizing modifiable risk factors for postoperative pain such as increased intra-articular pressure. Understanding that these factors may lead to increased pain after surgery can allow the healthcare team to better optimize pain management and patient counseling.

Our study faced several limitations that warrant consideration. First, the sample size was small, comprising only 52 patients. However, as previously stated, this study was explorative in nature, and therefore, a formal power analysis was not reasonable. Secondly, unlike Tan et al., we did not explore the impact of various fluid pump pressure settings on postoperative outcomes, potentially overlooking a significant risk factor for pain and opioid consumption. Also, postoperative intra-articular pressure experienced by patients may be different from the measured intra-articular pressure based on the thoroughness of joint evacuation following the procedure, which may affect patients’ reported VAS. Additionally, we did not correlate the pressure measurements of the STIC Intra-Compartmental Pressure Monitor System with measurements on the arthroscopic stack. However, the arthroscopic stack measurements would also need to be verified; hence, this may not provide any further benefit. While our study was prospective in nature, further analysis in the form of a randomized controlled trial may provide stronger data. Thirdly, reliance on the VAS introduced subjectivity and variability due to individual interpretations. Additionally, our assessment of opioid consumption was constrained by the inability to differentiate between patients who only partially used filled prescriptions and those who consumed all the medication. Fourthly, while efforts were made to standardize Stryker needling by employing a single operator, without confirmation of fluoroscopy, there is always room for error, making it challenging to replicate results in future studies. Finally, while the STIC Intra-Compartmental Pressure Monitor System produced by C2Dx has shown promising measures of accuracy in acute compartment syndrome, these findings should be further investigated to determine if they translate well to measuring intra-articular, and more specifically, glenohumeral joint pressure [29].

## Conclusions

These results suggest that an increased change in shoulder pressure is associated with a smaller VAS delta and less pain relief after surgery. Additionally, the greater change in pressure is associated with increased postoperative narcotics consumption. This emphasizes the importance of efficient surgical practices and controlled pressure management.
